# Rapid evolution of genetic and phenotypic divergence in Atlantic salmon following the colonisation of two new branches of a watercourse

**DOI:** 10.1186/s12711-017-0298-1

**Published:** 2017-02-14

**Authors:** Arne Johan Jensen, Lars Petter Hansen, Bjørn Ove Johnsen, Sten Karlsson

**Affiliations:** 1 Norwegian Institute for Nature Research (NINA), P.O. Box 5685, 7485 Sluppen, Trondheim, Norway; 2 Norwegian Institute for Nature Research (NINA), Gaustadalléen 21, 0349 Oslo, Norway

## Abstract

**Background:**

Selection acts strongly on individuals that colonise a habitat and have phenotypic traits that deviate from the local optima. Our objective was to investigate the evolutionary rates in Atlantic salmon (*Salmo salar*) in a river system (the Vefsna watershed in Norway), fewer than 15 generations after colonisation of two new branches of the watercourse for spawning, which were made available by construction of fish ladders in 1889.

**Methods:**

Differences in age and size were analysed using scale samples collected by anglers. Age and size of recaptures from a tagging experiment were compared between the three branches. Furthermore, genetic analyses of scale samples collected in the three river branches during two periods were performed to evaluate whether observed differences evolved by genetic divergence over this short period, or were the result of phenotypic plasticity.

**Results:**

We demonstrate that evolution can be rapid when fish populations are subjected to strong selection, in spite of sympatry with their ancestral group, no physical barriers to hybridisation, and natal homing as the only reproductive isolating barrier. After fewer than 15 generations, there was evidence of genetic isolation between the two branches based on genetic variation at 96 single nucleotide polymorphism loci, and significant differences in several life history traits, including size and age at maturity. Selection against large size at maturity appears to have occurred, since large individuals were reluctant to ascend the branch with less abundant water. The estimated evolutionary rate of change in life history traits is within the upper 3 to 7% reported in other fish studies on microevolutionary rates.

**Conclusions:**

These findings suggest that with sufficient genetic diversity, Atlantic salmon can rapidly colonise and evolve to new accessible habitats. This has profound implications for conservation and restoration of populations and habitats in order to meet evolutionary challenges, including alterations in water regime, whether altered by climate change or anthropogenic factors.

**Electronic supplementary material:**

The online version of this article (doi:10.1186/s12711-017-0298-1) contains supplementary material, which is available to authorized users.

## Background

Selection acts strongly on individuals that colonise a habitat and have phenotypic traits that are far from the local optima. Different environments may directly induce differences in the behaviour, morphology, and physiology of an individual [1, 2]. In most cases of natural colonisation, information on the source population and time since colonisation is unavailable. However, exotic species may be useful for observing evolutionary processes in real time [3, 4]. Rates of change have been measured for many such populations [5]; however, these metrics have been recorded often without examining whether this change is based entirely upon phenotypic plasticity, or if it involves genetic divergence [6, 7].

The introduction of salmonid fishes to new locations has provided the opportunity to study the rates and patterns of evolution [6, 8, 9]. Anadromous salmonids are known for their long feeding migrations at sea, where they gain most of their body mass, followed by returning, or homing, to their natal river to spawn. This is a general feature of Pacific salmon [10, 11] as well as Atlantic salmon [12, 13]. Homing is important for the evolution of local adaptive genetic characteristics in populations [14, 15]. Some straying may occur with the ability to colonise new habitats, and may—depending on the strength of selection—constrain local adaptation [16, 17]. Usually, individuals from donor populations have been stocked in new habitats isolated from their ancestors, and their rates have been estimated decades later, including chinook salmon (*Oncorhynchus tshawytscha*) in New Zealand [18–20], European grayling (*Thymallus thymallus*) in Norway [21–24], and sockeye salmon (*Oncorhynchus nerka*) in Lake Washington [8, 25]. Quantitative estimates are needed to determine how rapidly populations can evolve to meet new evolutionary challenges as a function of key population parameters [26]. Recent research has indicated that evolution can occur over relatively short (10 to 20 generations) periods of time [27]. For example, Hendry et al. [8] studied two adjacent sockeye salmon (*O. nerka*) populations in Lake Washington of common ancestry that colonised divergent reproductive environments (a river and a lake beach), and found evidence for the evolution of reproductive isolation after fewer than 13 generations.

In the current study, evolutionary rates for Atlantic salmon (*Salmo salar*) were investigated in a river system (the Vefsna watershed in Norway), fewer than 15 generations from the colonisation of two new branches of the watercourse for spawning, which were made available by construction of fish ladders in 1889. All Atlantic salmon in this river system occur in sympatry, with natal homing being the only barrier to genetic exchange between spawning areas. However, because the environmental conditions in these spawning areas differ considerably in terms of annual water flow and nutrients, we hypothesised that natural selection has been sufficient for the evolution of local genetic adaptation. Hence, life history, marine growth, size and age at maturity, and genetic divergence between Atlantic salmon from the two upper branches were compared to similar data from individuals captured in the main river further downstream (i.e., descendants of the ancestor population). Differences in age and size were analysed using scale samples collected by anglers between 1969 and 1981. Moreover, the age and size of recaptures in the three branches from a tagging experiment performed in 1979 were compared. Furthermore, genetic analyses of scale samples collected in the three river areas during two periods (1972 and 1979) were performed to evaluate whether observed differences evolved as the consequence of genetic divergence during this short period, or were entirely due to phenotypic plasticity.

## Methods

### Study site

**Fig. 1:**
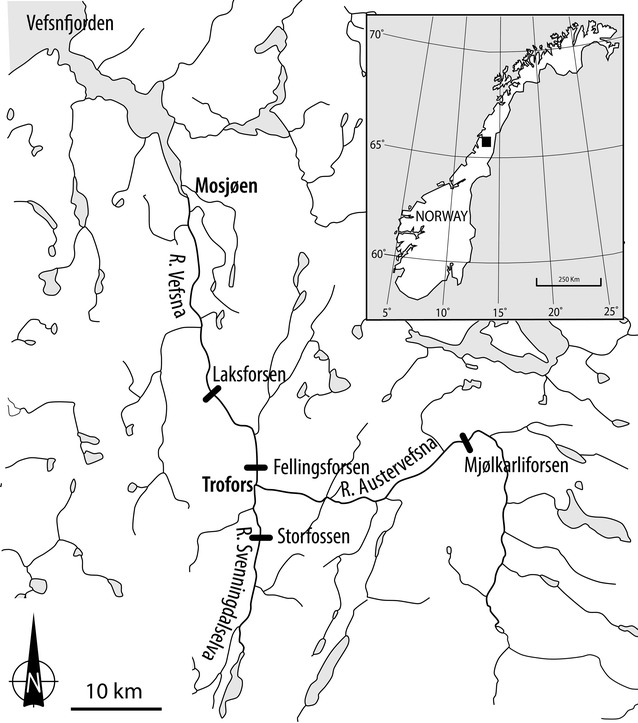
Map of the study area, including the Vefsna, Austervefsna, and Svenningdalselva rivers and waterfall locations

The Vefsna watershed is located in Norway in the northern reach of the Boreal Uplands [28]. The river mouth is located in the innermost part of the Vefsnfjorden fjord at 65°51′N, 13°11′E. The catchment area is 4231 km^2^ in size, and the mean annual water discharge at the outlet to the sea is 181 m^3^ s^−1^. Two main branches (the Austervefsna and Svenningdalselva rivers) meet at Trofors, 42 km from the sea. Downstream of this confluence, the name of the river changes to Vefsna. Starting at the Swedish border, the Austervefsna River flows mainly westwards to Trofors, where it merges with Svenningdalselva from the south, from where the name changes to Vefsna, and flows northwards to the sea at the city of Mosjøen (Fig. 1). Svenningdalselva has less water than Austervefsna, with mean annual discharges of 35 and 98 m^3^ s^−1^, respectively. The watercourse is rather steep, with several waterfalls, and an average gradient of 2.6 m km^−1^ [28]. The western part of the catchment (the Svenningdalen valley) consists of strongly transformed bedrocks from the Cambro-Silurian period, while the eastern catchment (Austervefsna) has little transformed bedrock from the same period, and a wide limestone belt. This influences the water quality, with higher values of total hardness, calcium, alkalinity, pH, and conductivity reported [28]. Hence, Austervefsna is slightly more productive than Svenningdalselva.

The predominant fish species at the study site are Atlantic salmon and brown trout (*Salmo trutta*). The Vefsna watershed used to be one of the largest inhabited by Atlantic salmon in Norway. In the late 1970s, however, the non-native parasite *Gyrodactylus salaris* was introduced to the river, and caused a severe decline in the Atlantic salmon population [29, 30]. Although the river was treated with rotenone in 2011 and 2012 to exterminate the parasite, and a recovery program was initiated to rebuild the Atlantic salmon population, this study is based on data collected before the invasion of the parasite, i.e., mainly during the 1970s.

Atlantic salmon reproduce in fresh water, where their offspring usually stay for 1 to 5 years before they migrate, at a length of 10 to 20 cm, to sea to feed and gain most of their weight. After 1 to 4 years at sea, they mature and return to their natal river with high precision, and even to the section of the river where they were born, to spawn [11, 31, 32]. The size of an individual at spawning, and hence its reproductive success, is dependent on the duration of its stay at sea (sea age). After one winter at sea, Atlantic salmon are typically 1 to 3 kg (50 to 65 cm), compared to 7 to 20 kg (>90 cm) after 3 years at sea [33].

Originally in the Vefsna watercourse, Atlantic salmon could only ascend to the Laksforsen waterfall, about 29 km from the sea. In 1889, fish ladders were established in the 16-m high Laksforsen and in another waterfall (Fellingforsen, 5-m high) further upstream in Vefsna. This provided access to the upper parts of Vefsna, and a further 24 and 4 km upstream, the Austervefsna (to the Mjølkarliforsen waterfall) and Svenningdalselva (to the Storfossen waterfall) rivers, respectively [34]. In 1903, another 23 km of the Svenningdalselva River was opened for salmon, when a new fish ladder was constructed in the 7-m high Storfossen waterfall [34]. In 1922, fish ladders were built in four different waterfalls further upstream in the Austervefsna River, including Mjølkarliforsen, allowing salmonids to migrate a further 22 km upstream. In addition, particularly in the 1950s, old fish ladders were improved and new ladders were constructed in Austervefsna [34]. Hence, today, 126 km of the watercourse up to 330 m above sea level is accessible to this species. All ladders are of the same type (pool and weir ladders), and all new stretches have been colonised naturally, i.e., without stocking of fish.

### Age and growth

Age and growth were analysed from scale samples of adult Atlantic salmon collected by anglers during the period between 1969 and 1981. In total, 3089 samples were available, of which 2060, 595, and 434 were collected in Vefsna, Austervefsna, and Svenningdalselva, respectively (Table 1; Additional file 1: Table S1). Information on natural tip length (i.e., total length with the caudal fin in natural position), mass, sex, catch date, and location were recorded for each individual. Sex was determined by the anglers through external inspection of the fish. Individuals identified from their scales as multiple spawners (i.e., spawned more than once) were excluded from further analyses (0.7%).

**Table 1:** Number of scale samples from Atlantic salmon collected in the three river stretches, sorted by collection year

Year	Vefsna	Austervefsna	Svenningdalselva	Total
1969	5	0	40	45
1971	4	4	69	77
1972	148	52	50	250
1973	240	51	55	346
1974	52	187	126	365
1975	104	29	19	152
1977	305	0	0	305
1978	308	109	15	432
1979	375	63	46	484
1980	283	59	9	351
1981	236	41	5	282
Total	2060	595	434	3089

Linear mixed models, with year as a random factor, were used to test similarity in fish length and smolt age between individuals captured in the three river branches [35].

The evolutionary rate of change (i.e., phenotypic change in standard deviations per generation [36]) in length, mass, and sea age of Atlantic salmon in the two upper branches was measured in haldanes (*h*), calculated as:

$$h = \frac{{\left( {x_{2} /S_{p} } \right) - \left( {x_{1} /S_{p} } \right)}}{g},$$

where, $x_{1}$ is the mean natural log measurements from either the Svenningdalselva or Austervefsna, and $x_{2}$ is the corresponding measurement from the Vefsna River; *g* is the number of generations since the upper branches of the watercourse were opened to anadromous fish (i.e., since 1889), and $S_{p}$ is the pooled standard deviation (SD) from the natural log measurements for both populations [6]. Data from Vefsna prior to establishing the first fish ladders in 1889 were not available; therefore, corresponding data obtained from samples collected in the part of Vefsna where anadromous fish were naturally distributed (i.e., downstream from the Laksforsen waterfall) between 1969 and 1981 were used as a proxy. This assumption may lead to some uncertainties regarding evolutionally rates per se [6]. Some individuals from the two upper branches were likely to have been included in the catches in the lower part of the watercourse; hence, underestimation of mean length, mass, and sea-age of Atlantic salmon in the lower part of the Vefsna, and, by extension, evolutionary rates of change (in *h*), is probable.

### Tagging experiment

In the period from 25 June to 20 September 1979, 1130 adult Atlantic salmon were captured in the fish ladder in the Laksforsen waterfall during their migration upstream, individually tagged with Lea tags, and released again. During tagging, the water temperature varied between 7 and 16 °C. Most individuals were tagged in July at temperatures between 11 and 16 °C. In a note included in each Lea tag, the angler was asked to report the length and mass of the fish, the day and exact locality of recapture, and a scale sample was requested (see Additional file 2: Table S2). Individuals recovered in Austervefsna and Svenningdalselva were assumed to have homed, whereas those recovered in Vefsna might also belong to the upper sections.

### Genetic analyses

Overall, 285 individuals, 95 from each of Austervefsna, Svenningdalselva, and Vefsna, were assayed for genetic variation at 96 single nucleotide polymorphisms (SNPs). Half of the samples were collected in 1972 and the remainder were collected in 1979. Eighteen individuals had a genotyping success lower than 80% and were excluded from further analyses. The remaining 267 successfully genotyped individuals were included in analyses of temporal and spatial genetic variation (see Additional file 3: Table S3).

Total genomic DNA was extracted from dried scale samples using the DNeasy kit from Qiagen (Hombrechtikon, Switzerland). Ninety-six SNPs previously described by Bourret et al. [37] were genotyped with an EP1™ 96.96 Dynamic array IFCs (Fluidigm, San Francisco, CA, USA). Fifteen of the 96 SNPs were located within the mitochondrial genome [38].

Observed and expected heterozygosity levels at the 81 nuclear SNPs within individuals from the Austervefsna, Svenningdalselva, and Vefsna rivers were estimated using GENALEX 6.0 [39], and deviation from Hardy–Weinberg equilibrium was tested in Genepop v. 4.1.4 [40]. The 15 SNPs in the mitochondrial DNA (mtDNA) were compiled into haplotypes, and standard genetic indices, including number of haplotypes, haplotype diversity, and nucleotide diversity, were estimated in Arlequin version 3.5 [41].

Estimates of pairwise *F*_ST_ and tests for differences in allele frequencies between samples from Austervefsna, Svenningdalselva, and Vefsna sampled in 1972 and 1979 were performed in Genepop v. 4.1.4 [40]. Genetic differences between sampling localities and sampling years were visualised in a principal coordinate analysis (PCoA) plot based on pairwise *F*_ST_ as implemented in GENALEX 6.0 [39]. Estimates of pairwise *F*_ST_ and tests for significance variance in the mitochondrial SNPs were performed in Arlequin [41]. To investigate possible migrants, individual genetic assignment to the three branches of the watershed, so called self-assignment, was conducted using the direct assignment approach and the Bayesian method [42], as implemented in GeneClass2 [43]. Relatedness coefficients between individuals were estimated using the Coancestry program [44], and the level of relatedness between individuals within Austervefsna was compared against that within Svenningdalselva using the same program.

Effective population size $(N_{e} )$ was estimated using the linkage disequilibrium (LD) method [45, 46] implemented in LDNE [47] and the temporal method with Fs estimator implemented in the TempoFS software [48], applying sample plan 2.

## Results

### Age and growth

**Fig. 2:**
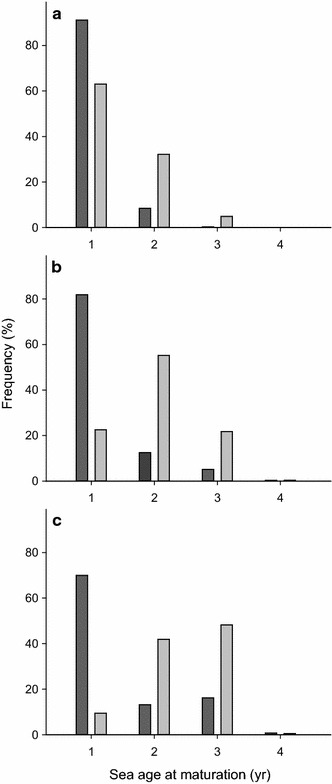
Sea age distribution (%) of males (*dark grey*) and females (*light grey*) in **a** Svenningdalselva, **b** Austervefsna, and **c** Vefsna. Data were obtained from scale samples of Atlantic salmon collected between 1969 and 1981. Previous spawners are excluded from the material

As for most Atlantic salmon populations containing multi-sea-winter (MSW) fish, sea age at maturity in the Vefsna watercourse differed between sexes, with a majority of males returning after one winter at sea (1SW), and females after two or three winters at sea (2SW and 3SW, respectively). Sea age at maturity differed; however, among the three river stretches, with the youngest individuals (both males and females) found in Svenningdalselva and the oldest in Vefsna (Fig. 2). The mean sea age (±95% confidence interval, CI) of males captured in Svenningdalselva was 1.10 ± 0.04 years, while the corresponding means in Austervefsna and Vefsna were 1.24 ± 0.06 and 2.10 ± 0.10 years, respectively. Females were usually older than males (one-way ANOVA; Svenningdalselva: F_1,429_ = 59.7, P < 0.001; Austervefsna: F_1,602_ = 217.4, P < 0.001; Vefsna: F_1,1991_ = 806.9, P < 0.001), with mean sea ages of 1.47 ± 0.10, 2.01 ± 0.08, and 2.55 ± 0.04 years in Svenningdalselva, Austervefsna, and Vefsna, respectively. The sex ratio differed significantly between Vefsna and the two other river stretches (X^2^ = 1563, P < 0.001), since males predominated in Svenningdalselva and Austervefsna (62 and 57%, respectively), while females comprised the majority of salmon caught in Vefsna (53%).

The length of Atlantic salmon differed significantly between the three river branches (one-way ANOVA, F_2,3468_ = 173.4, P < 0.001), with the smallest individuals captured in Svenningdalselva (mean length 613.7 ± 10.7 mm, *n* = 433), intermediate-sized individuals were caught in Austervefsna (714.3 ± 12.6 mm, *n* = 595), and the largest individuals were caught in Vefsna (765.2 ± 7.7 mm, *n* = 2046), partly because of differences in sea age when they returned to the river. When grouping by sea age, the mean lengths of both 1SW and 2SW fish remained the smallest in Svenningdalselva, both among males and females (Table 2). Controlling for sex, sea age, and river branch, and with year as a random factor in a linear mixed model, individuals captured in Svenningdalselva were significantly smaller than in both Austervefsna (−25.5 ± 8 mm, t = 6.18, *df* = 157, P < 0.001) and Vefsna (−20.6 ± 8.8 mm, t = 4.61, *df* = 1413, P < 0.001).

**Table 2:** Mean length (mm ± 95% CI) of adult Atlantic salmon captured in three river stretches of the Vefsna watercourse between 1969 and 1981, maturing after one, two, and three winters at sea, respectively, and sorted between males and females

River	Vefsna	Austervefsna	Svenningdalselva
*Males*			
1SW	589.8 ± 9.0 (118)	592.1 ± 5.1 (261)	573.3 ± 5.9 (205)
2SW	848.3 ± 11.2 (97)	849.1 ± 29.5 (41)	777.4 ± 44.4 (23)
3SW	1003.5 ± 11.9 (138)	951.7 ± 41.0 (17)	NA
*Females*			
1SW	575.0 ± 25.3 (17)	575.0 ± 13.3 (52)	560.5 ± 9.1 (85)
2SW	809.3 ± 5.1 (345)	827.0 ± 11.7 (135)	794.2 ± 19.4 (52)
3SW	928.2 ± 4.4 (476)	952.7 ± 12.2 (54)	945.0 ± 34.3 (8)

Individuals identified as previous spawners were excluded. Sample sizes are in parentheses

*1SW* one sea winter, *2SW* two sea winters, *3SW* three sea winters, *NA* not available

The mean (±SD) age of adult Atlantic salmon when they returned to the watercourse as mature individuals, was 5.68 ± 1.04 years; they stayed 3.91 ± 0.65 years in fresh water (smolt age) until they migrated to sea, and remained 1.78 ± 0.83 years at sea (sea age at maturity) before they returned to the river to spawn. The mean length of adult fish after a stay at sea of 1, 2, and 3 years was 568 ± 46, 820 ± 67, and 947 ± 61 mm, respectively.

The predominating smolt age was 4 years (2- to 6-year range) in all three branches of the watercourse. The mean smolt age (±95% CI) was greater in Svenningdalselva (3.97 ± 0.06 years, *n* = 420) than in Austervefsna (3.88 ± 0.05 years, *n* = 580) and Vefsna (3.90 ± 0.03 years, *n* = 1838). Controlling for sex and river branch, and with year as a random factor in a linear mixed model, individuals captured in Svenningdalselva were significantly older when they migrated to sea as smolts than in both Austervefsna (0.10 ± 0.09 year, t = −2.13, *df* = 886, P < 0.05) and Vefsna (0.20 ± 0.09 year, t = −4.39, *df* = 1659, P < 0.001).

Estimated evolutionary rates of change in the length, mass, and sea age of Atlantic salmon between 1889 and the 1970s, were higher in the Svenningdalselva population than in the Austervefsna population, and higher for females than for males, with values varying between −0.054 and −0.075 *h* for Austervefsna and −0.086 to −0.187 *h* for Svenningdalselva (Table 3).

**Table 3:** Evolutionary rate of change in haldanes (*h*) in length, mass, and sea age at maturity in male and female Atlantic salmon estimated from scale samples collected between 1969 and 1981

River branch	Length (*h*)		Mass (*h*)		Sea age (*h*)	
	Males	Females	Males	Females	Males	Females
Austervefsna	−0.060	−0.054	−0.075	−0.071	−0.075	−0.074
Svenningdalselva	−0.086	−0.161	−0.106	−0.187	−0.094	−0.162

Data obtained from scale samples collected in the lower part of Vefsna (where anadromous fish were naturally distributed, i.e., downstream of the Laksforsen waterfall) were used as a proxy for corresponding data prior to the first fish ladder being constructed in 1889

### Tagging experiment

**Fig. 3:**
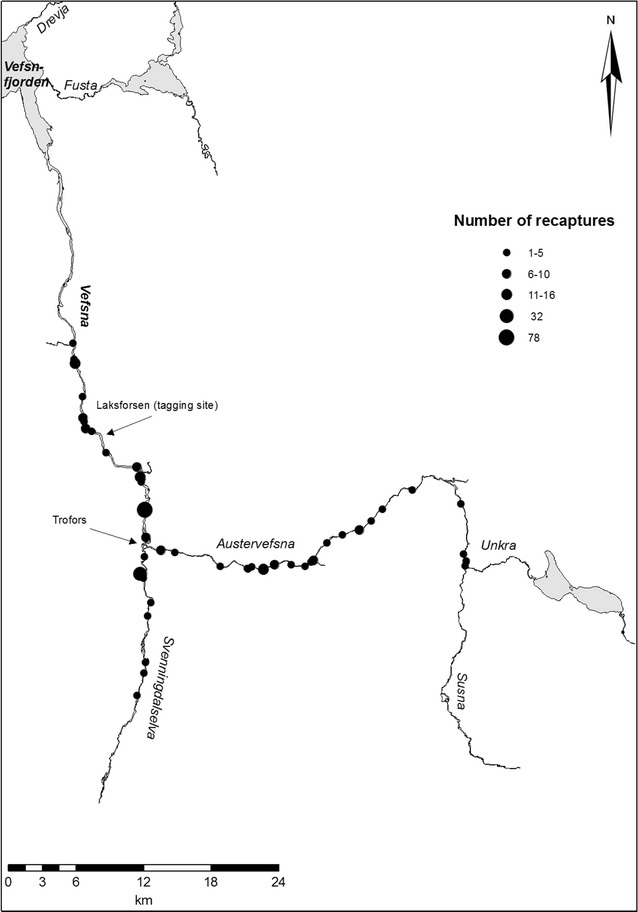
Geographic distribution of adult Atlantic salmon recaptures within the Vefsna watercourse tagged in the Laksforsen waterfall during 1979

**Fig. 4:**
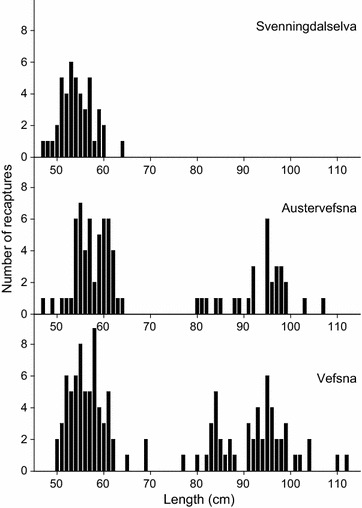
Length distribution of Atlantic salmon tagged at the Laksforsen waterfall in 1979 and recaptured in different branches of the Vefsna watercourse the same year. Lengths are based on the measurements at tagging

Among the 315 recaptures reported in the same year that they were tagged, 82 were reported in Austervefsna, 44 in Svenningdalselva, and 117 in Vefsna, upstream of the tagging location (Fig. 3). The length distribution of individuals recaptured in Svenningdalselva differed from that of individuals captured from the two other river stretches (Fig. 4). In Svenningdalselva, only individuals less than 650-mm long were caught, while in the two other river branches, a significant portion of the catch was larger (between 800 and 1100 mm in length). Such patchy length distributions reflect different sea age groups, where individuals smaller than 700 mm from this watercourse stayed for only one winter at sea, while larger individuals were a mixture of 2SW, 3SW, and 4SW fish (Fig. 4). Thus, recaptures smaller than 700 mm were assumed to be 1SW fish, and these were significantly smaller in Svenningdalselva compared within Austervefsna [mean lengths 541.8 ± 10.5 (±95% CI) mm and 572.3 ± 9.8 mm in Svenningdalselva and Austervefsna, respectively (one-way ANOVA: F_1,95_ = 18.09, *P* < 0.001)]. The mean length of 1SW fish recaptured in Vefsna upstream of the tagging location was 567.9 ± 7.3 mm, i.e., intermediate between that of the two other river stretches (significantly larger than that in Svenningdalselva [one-way ANOVA: F_1,147_ = 15.43, *P* < 0.001], but not significantly different from that in Austervefsna [one-way ANOVA: F_1,156_ = 0.49, *P* < 0.05]). No MSW fish from the tagging experiment was captured in Svenningdalselva, while 30 individuals (36%) captured in Austervefsna and 59 individuals (36%) captured in Vefsna were MSW fish.

### Genetic analyses

No significant deviations from Hardy–Weinberg equilibrium were detected within individuals from the Austervefsna, Svenningdalselva, and Vefsna rivers, and the average observed and expected heterozygosities in the three localities were 0.332 to 0.352 and 0.332 to 0.339, respectively, with the lowest level in Svenningdalselva. Svenningdalselva also had fewer mitochondrial haplotypes (4), and the lowest levels of nucleotide diversity (π = 0.102) and haplotype diversity (hd = 0.446) compared to Vefsna (six haplotypes, π = 0.144, and hd = 0.167).

**Fig. 5:**
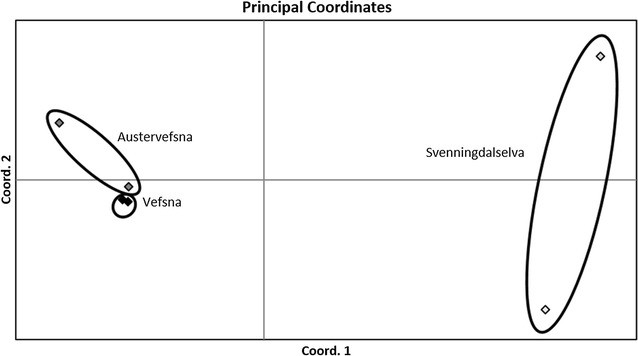
Principal coordinate analysis (PCoA) plot from pairwise *F*_ST_ estimates, based on 81 SNPs, between samples from Austervefsna (*grey diamonds*), Svenningdalselva (*white diamonds*), and Vefsna (*black diamonds*) collected in 1972 and 1979. The first and second axes explain 34 and 23% of the genetic variance, respectively

**Fig. 6:**
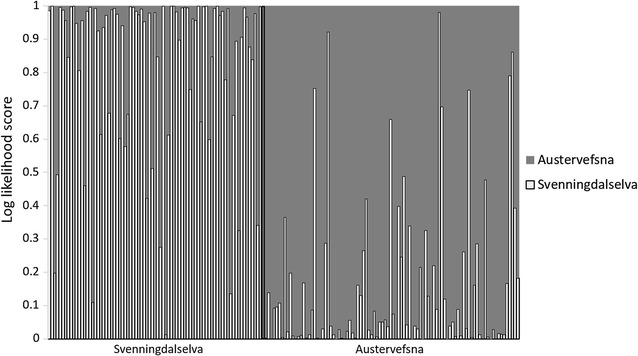
Individual genetic assignment of Atlantic salmon from Svenningdalselva and Austervefsna to Svenningdalselva and Austervefsna, i.e., self-assignment. For each individual, the relative probability of assignment (log likelihood score) to Austervefsna (*grey bars*) and Svenningdalselva (*white bars*) is given on the y axis

There were no significant genetic differences between sampling years within localities (*P* > 0.305). Based on 81 nuclear SNPs, a temporally stable genetic structure was observed (Fig. 5); the subpopulation from Svenningdalselva was significantly different (P < 0.001) from those from Austervefsna and Vefsna, with estimated *F*_ST_ values of 0.012 and 0.011, respectively. The subpopulation from Austervefsna was not significantly different from that from Vefsna (*F*_ST_ = −0.001, *P* = 0.802). Genetic variation at 15 mitochondrial SNPs revealed that fish collected at Svenningdalselva were significantly genetically distinct from those in the Vefsna (*F*_ST_ = 0.038, *P* < 0.001), but not from those in the Austervefsna (*F*_ST_ = −0.008, *P* = 0.766). In terms of the genetic assignment of individuals from the Austervefsna and Svenningdalselva rivers, 8.5% of the individuals from Austervefsna were assigned to Svenningdalselva and not to Austervefsna, while 12.7% of the individuals from Svenningdalselva were assigned to Austervefsna and not Svenningdalselva (Fig. 6). This suggests an exchange of individuals, and possible gene flow between Austervefsna and Svenningdalselva, with a higher rate of gene flow observed from Austervefsna to Svenningdalselva than from Svenningdalselva to Austervefsna.

According to the Wang estimator, average relatedness between individuals from Svenningdalselva was 0.017, and was significantly higher at the 2.5 percentile than that between individuals from Austervefsna (−0.010). This finding, combined with the lower genetic variation and significant genetic differentiation of fish in the Austervefsna and Vefsna branches, indicates the presence of a lower effective population size $(N_{e} )$ in Svenningdalselva compared to Austervefsna and Vefsna.

Estimates of $N_{e}$ using the temporal method were 71 in Svenningdalselva, with a 95% confidence interval (CI) from 31 to $\infty$, while no reasonable estimates were obtained for Austervefsna and Vefsna (negative values). A negative value indicates that most of the observed shift in allele frequency is explained by the sampling variance and not by the genetic drift, and that large sample sizes are needed [47, 49]. In addition, temporal samples were only one generation apart, which also lowers the precision of this method. The LD method for estimating current $N_{e}$ gave reasonable estimates (with 95% CI) for Austervefsna 1972, 171 (94–656); Svenningdalselva 1979, 140 (79–472); Vefsna 1972, 249 (123–3210); and Vefsna 1979, 1931 (221–$\infty$).

## Discussion

In accordance with previous studies [5, 8, 50, 51], the findings reported here demonstrate that evolution can occur rapidly when fish populations encounter strong selection. In addition, this can occur even in sympatry with an ancestral population, isolated by natal homing only, and with no physical barriers to hybridisation. In the Vefsna watercourse, strong selection for smaller and younger individuals at maturity may be due to the lower water discharge in the two upper branches compared to the main river further downstream, and hence reluctance of the largest individuals to ascend these branches, particularly the branch that has less water (Svenningdalselva).

An alternative, albeit less likely, hypothesis to explain the smaller size of Atlantic salmon ascending Svenningdalselva compared with that from the other river stretches could be difficulties for MSW fish in crossing the fish ladder in Storfossen in Svenningdalselva. However, with a fall of 7 m, Storfossen is not considered more difficult to cross than the other fish ladders in the watercourse. Furthermore, this fall is located 4 km upstream from the confluent with Austervefsna, and the lower 4 km stretch of Svenningdalselva, which was already open for Atlantic salmon in 1889, is as suited to produce young fish as the remainder of the watercourse.

Atlantic salmon captured in the two upper branches, which were both made available from 1889 following fish ladder construction in Vefsna, were a genetically distinct lineage with unique population demographics, including smolt age, and sea age and size at maturity. In both river branches, sea age and size at maturity were lower than those in the main river further downstream. Individuals captured in the Svenningdalselva section genetically diverged from those captured in the other two river branches; in addition, individuals of the same sea age were smaller. Both upper branches had been available for Atlantic salmon for a maximum of 83 years before the first genetic sample was collected (in 1972), which corresponds to fewer than 15 generations.

In this study, large individuals appeared reluctant to ascend Svenningdalselva, and some also hesitated to ascend Austervefsna due to the lower level of water compared to the downstream of their confluence. In Atlantic salmon, there is a large increase in mass due to each additional summer at sea, compared to the variance in mass among fish of the same sea age. Hence, this selection pressure also affects duration at sea and age at maturity. In general, small rivers contain smaller and younger Atlantic salmon than larger rivers [52]. In a study of Atlantic salmon in 18 Norwegian rivers, mean body length and mean sea age at maturity increased with increasing annual discharge of the spawning river until 40 m^3^ s^−1^, while at higher discharges, no such correlation was found [53]. Jonsson et al. [53] argued that selection in such rivers may act against large salmon because of low water flow, which increases the risk of successful ascent and breeding by large salmon, counteracting size advantages such as fecundity and competitive ability. This study supports the hypothesis of Jonsson et al. [53], who proposed that body size and sea age at maturity increase with increasing annual discharge. From both Austervefsna and Svenningdalselva, lower values were found for these life history traits compared with those from Vefsna, suggesting that Atlantic salmon with larger body sizes may also hesitate to ascend rivers at discharges higher than 40 m^3^ s^−1^.

Mean smolt age was greater in Svenningdalselva than in Austervefsna, demonstrating high precision homing to each river branch. Growth differences of juvenile parr—and hence, differences in smolt age—are expected to be caused by the slightly higher productivity in Austervefsna than in Svenningdalselva, because of differences in geology and hence water quality [28]. Water temperature may also have affected growth [54], although data on the temperature regimes of these branches are not available.

Atlantic salmon from Svenningdalselva have diverged from their source population in the lower part of Vefsna, presumably because of interplay between adaptation and ecologically-mediated reproductive isolation [55]. Because of the precise homing behaviour of salmonids to the river section where they are born, colonising habitats upstream of new fish ladders might be a slow process, depending mainly on strayers from downstream stretches or other rivers [17, 56]. After their first sea sojourn, offspring of these strayers are expected to ascend to the location of the river where they were born to spawn. To the upper stretches of the Vefsna watershed, most strayers are expected to belong to downstream sections of the Vefsna watershed, rather than to other rivers. This is because Vefsna housed the largest Atlantic salmon population within 200 km before the fish ladders were constructed, with reported annual catches in the 1880s of 1.5 to 4 tons [57].

The Atlantic salmon population in the Vefsna watercourse is a multi-sea-winter (MSW) population, with a high proportion of large individuals, such that approximately 35% of individuals captured in Vefsna (i.e., downstream Trofors) were longer than 900 mm, and had stayed three or more winters at sea, with a predominance of females. Results from both the tagging experiment and catches in different parts of the watercourse demonstrate that most of the large individuals (3SW or older) were captured in Vefsna. Furthermore, males comprised more of the population in Svenningdalselva and Austervefsna, whereas females predominated in Vefsna. These results suggest that large individuals (predominately females) born in the two upper sections hesitated to ascend these sections, and hence stayed in Vefsna for longer than their smaller conspecifics. In this way, the probability of being caught by anglers in Vefsna was higher than that for their smaller conspecifics, and may have resulted in a predominance of females in catches from Vefsna, and a predominance of males further upstream. This suggests that there is strong selection for smaller body size of fish in the two upper branches, especially in the section with the lowest water flow (Svenningdalselva). This hypothesis is supported by the differences observed between Svenningdalselva and Austervefsna in nuclear and mitochondrial SNPs. Significant differences are found between Svenningdalselva and Austervefsna in nuclear SNPs, but no such differences are observed in maternally inherited mtDNA, despite an expected higher evolutionary rate (lower effective population size) in mtDNA. This indicates a higher rate of gene flow in females than in males. The age and size at which an individual reproduces is an important life history trait. In Atlantic salmon, body size (and hence the number of years spent at sea before maturity) is important for reproductive success, and is more important for females than for males [58]. There is evidence for sex-specific selection patterns on sea age at maturity, as life-history strategies differ considerably between females and males [58]. A recent discovery that a single locus controls up to 39% of the variation in age at maturity in Atlantic salmon in a sex-specific manner [59] substantiates such rapid selection for adaptation in size-related traits under a changing environment.

The estimated evolutionary rates for life history traits in this study were in the upper range of values (for Svenningdalselva the upper 3 to 7%) found in other fish studies reporting microevolutionary rates [5]. Furthermore, they were similar to the highest rates observed in fish in response to stream impoundment [60], but lower than those for exploited fish stocks [61, 62]. Genetic differences in this study appeared within fewer than 15 generations, which is similar to the 10 to 20 generations reported as the shortest time observed for other vertebrates [27].

## Conclusions

The results of this study suggest that with sufficient genetic diversity, Atlantic salmon can rapidly colonise and evolve to new habitats if made accessible. This has profound implications for conservation and restoration of populations and habitats to meet evolutionary challenges, including alterations in water regime, whether in response to climate change or anthropogenic factors.

## Additional files

**Additional file 1: Table S1.** Information on scale samples for length, mass, sex, smolt age, and sea age of adult Atlantic salmon captured in different sections of the Vefsna watershed during the period 1969 to 1981.

**Additional file 2: Table S2.** Recaptures of adult Atlantic salmon from a tagging experiment performed in Vefsna in 1979. Only recaptures upstream of the tagging site during the year of tagging are included.

**Additional file 3: Table S3.** Genotype data for 96 SNPs in Atlantic salmon from the Vefsna watershed.
